# Fréquence élevée de l´ischémie myocardique asymptomatique dans une population de patients infectés par le VIH à Bobo-Dioulasso, Burkina Faso

**DOI:** 10.11604/pamj.2021.38.173.9509

**Published:** 2021-02-16

**Authors:** Jean-Baptiste Tougouma, Arsène Aimé Yaméogo, Nobila Valentin Yaméogo, Arsène Hema, Hervé Hien, Yibar Kambiré, Armel Poda, Jacques Zoungrana, Patrice Zabsonré

**Affiliations:** 1 Institut Supérieur des Sciences de la Santé (INSSA), Université Polytechnique de Bobo Dioulasso (UPB), Bobo Dioulasso, Burkina Faso,; 2 Unité de Formation et de Recherche en Sciences de la Santé, Université de Ouagadougou, Ouagadougou, Burkina Faso,; 3 Centre Hospitalier Universitaire Sourô Sanou (CHUSS), Bobo Dioulasso, Burkina Faso,; 4 Centre Muraz, Bobo-Dioulasso, Burkina Faso

**Keywords:** Ischémie myocardique asymptomatique, VIH, traitement ARV, Burkina Faso, Asymptomatic myocardial ischemia, HIV, ARV therapy, Burkina Faso

## Abstract

**Introduction:**

les complications cardiovasculaires sont devenues la 3^e^ cause de décès et le 4^e^ motif d´hospitalisation des patients infectés par le VIH. L´objectif de ce travail était de déterminer la fréquence de l´ischémie myocardique asymptomatique chez les patients infectés par le VIH sous traitement antirétroviral.

**Méthodes:**

étude transversale descriptive qui s´est déroulée en novembre 2015. Ont été inclus les patients infectés par le VIH1, sous traitement ARV, asymptomatiques suivis dans l´unité d´Hôpital de Jour du service des Maladies Infectieuses du CHU Souro Sanou de Bobo-Dioulasso. Les patients inclus ont bénéficié d´un recueil des facteurs de risque cardiovasculaire, de deux mesures en consultation de la pression artérielle en position assise après 10 minutes de repos et d´un électrocardiogramme 12 dérivations de repos.

**Résultats:**

au total, 123 patients infectés par le VIH1 avec un âge médian de 42 ans (IIQ: 36-50) et composés à 79% de sujets de sexe féminin ont été inclus. Les facteurs de risque cardiovasculaire retrouvés se répartissaient comme suit: HTA (31,7%), obésité (33%), dyslipidémies (10,57%), tabagisme actif (0,8%), diabète (0,8%). Tous les hypertendus connus (5,7%) étaient insuffisamment traités. La durée médiane d´exposition aux ARV était de 5,3 années (IIQ: 3-7,7). Les troubles de la repolarisation étaient observés dans 26 cas (21,13%). Ils se répartissaient en ischémie sous épicardique dans 20 cas (16,26%), lésion sous endocardique dans 2 cas (1,63%) et en séquelle de nécrose dans 4 cas (3,25%). L’hypertrophie ventriculaire gauche (HVG) était observée dans 12 cas (9,76%) et était observée chez les patients hypertendus. Le QTc était allongé chez 7 patients (5,69%) indépendamment de la classe d´ARV administré.

**Conclusion:**

dans cette étude sur les patients infectés par le VIH1, il ressort que l´ischémie myocardique asymptomatique est fréquente. Il conviendrait de renforcer son dépistage par des tests d´ischémie plus performants afin de mieux préciser sa gravité dans cette sous population au risque cardiovasculaire accru.

## Abstract (en)

**Introduction:**

cardiovascular complications have become the 3^th^ cause of death and the 4^th^ reason for hospitalization in HIV-infected patients. The purpose of this study was to determine the frequency of asymptomatic myocardial ischemia in HIV-infected patients on antiretroviral therapy.

**Methods:**

we conducted a descriptive cross-sectional study in November 2015. Asymptomatic HIV-1-infected patients on ARV treatment and followed up in the Day Hospital Unit of the Department of Infectious Diseases of the University Hospital Sanon Sourou of Bobo-Dioulasso were included in the study. Among enrolled patients data on cardiovascular risk factors were collected as well as two sitting blood pressure measurements after 10 minutes of rest were taken during consultations and resting 12-lead electrocardiogram (ECG) was performed.

**Results:**

a total of 123 HIV-1-infected patients with a median age of 42 years (IQR: 36-50), among whom 79% were female subjects, were included in the study. Cardiovascular risk factors included: PAH (31.7%), obesity (33%), dyslipidemia (10.57%), active smoking (0.8%) and diabetes (0.8%). All patients with hypertension (5.7%) were insufficiently treated. The median duration of ARV treatment was 5.3 years (IQR: 3-7.7). Repolarization disorders were found in 26 cases (21.13%). They were divided into subepicardial ischaemia in 20 cases (16.26%), subendocardial damage in 2 cases (1.63%) and sequelae of necrosis in 4 cases (3.25%). Left ventricular hypertrophy (LVH) was found in 12 cases (9.76%) and, in particular, in hypertensive patients. Prolonged QTc interval was found in 7 patients (5.69%) regardless of the ARV drugs given.

**Conclusion:**

this study of HIV-1-infected patients highlights that asymptomatic myocardial ischemia is common. Screening programmes should be improved through more effective ischemia tests in order to better determine its severity in this sub-population with increased cardiovascular risk.

## Introduction

Le risque cardiovasculaire des patients infectés par le virus de l´immunodéficience humaine (VIH) va devenir dans les années futures, une préoccupation de plus en plus importante pour les médecins prenant en charge cette infection en raison du succès du traitement antirétroviral (ARV) entraînant un allongement considérable de la survie des patients infectés mais aussi des effets secondaires de ce même traitement. Depuis l´apparition du traitement antirétroviral efficace (HAART), le spectre de l´atteinte cardiologique au cours de l´infection par le VIH a été modifié. Il s´agit maintenant de faire face aux complications liées au traitement antirétroviral, en particulier l´accélération de l´athérosclérose (maladie coronaire, artériopathie des membres inférieurs, accident vasculaire cérébral) [1-3]. Les études épidémiologiques ont montré que l´incidence de l´infarctus du myocarde est plus élevée dans la population VIH que dans la population générale [4, 5]. En Afrique la fréquence de la maladie coronaire chez le patient infecté par le VIH est mal connue et quelques rares cas sont rapportés dans des séries hospitalières [6, 7]. La maladie coronaire à l´instar d´autres maladies métaboliques (diabète) peut être asymptomatique et de découverte fortuite. Cette étude avait pour objectif de décrire la fréquence de l´ischémie myocardique asymptomatique dans une population de patients infectés par le VIH sous traitement antirétroviral.

## Méthodes

L´étude a été réalisée dans le service de l´hôpital du jour (HDJ) du département de médecine du CHU Sanou Sourô (CHU SS) de Bobo-Dioulasso.

Il s´agissait d´une étude transversale descriptive qui s´est déroulée du 2 au 30 novembre 2015. La population d´étude était celle des patients suivis en routine dans la cohorte des patients infectés par le VIH de l´HDJ du CHUSS. C´est une cohorte constituée progressivement depuis 1998. Les patients inscrits dans cette cohorte sont au nombre de 5000. L´échantillonnage était raisonné. C´était un recrutement consécutif des patients infectés par le VIH sur la période d´étude et répondant aux critères d´inclusion.

Ont été inclus dans cette étude les patients infectés par le VIH1, sous traitement ARV, et ayant donné leur consentement.

**Collecte des données:** les patients inclus ont bénéficié: d´un recueil des facteurs de risque cardiovasculaire (âge, obésité, tabac, contraception orale); les paramètres liés à l´infection par le VIH (date de découverte de la séropositivité, stade SIDA, nadir CD4, taux de CD4, charge virale dans les 3 mois précédents, le traitement antirétroviral actuel); de deux mesures en consultation de la pression artérielle en position assise après 10 minutes de repos; et d´un électrocardiogramme de surface de repos 12 dérivations permettant l´étude du rythme, de la conduction et de la repolarisation.

Les définitions suivantes ont été utilisées: patients hypertendus étaient définis par une PAS ≥ 140 mmHg et/ou PAD ≥ 90 mmHg en consultation et/ou sous traitement antihypertenseur quelle que soit la régularité du traitement; l´ischémie sous épicardique était définie par la présence d´ondes T négatives concordantes dans un territoire coronaire; la lésion sous endocardique était définie par un sous décalage significatif du segment ST (> 1,5mm) concordant dans un territoire coronaire; la séquelle de nécrose était définie par une abrasion des ondes R concordantes dans un territoire coronaire et/ou une onde Q large (≥ 4/100^e^ sec) et profonde (≥ 1/10^e^ mv); l´hypertrophie ventriculaire gauche (HVG) était définie par un indice de Sokolov-Lyon ≥ 35mm; l´allongement de l´espace QT était défini par un QT calculé selon la formule de Bazett ≥ 0,44s.

Le mode de collecte de données: nous avons constitué une équipe de cliniciens (infectiologue et cardiologue) déjà investis dans le suivi de la cohorte de ces patients pour collecter les données pendant la période d´étude.

Les données saisies sous Epidata, ont été analysées grâce au logiciel STATA® 13. Les caractéristiques sociodémographiques, cliniques, biologiques et électrocardiographiques des patients ont été décrites. Les variables quantitatives ont été décrites par les médianes (me) et leurs interquartiles (IIQ) et les variables qualitatives par leur proportion (%). Nous avons utilisé le test exact de Fisher pour la comparaison des données qualitatives et le test t de Student pour celle des données quantitatives.

Les données ont été collectées avec le consentement éclairé et écrit des patients. Une notice d´information et un formulaire de consentement ont été introduits auprès des patients pour recueillir leur consentement. Des autorisations des responsables de la cohorte et du Département de médecine ont été obtenues avant le début de la collecte des données.

## Résultats

Cent vingt-trois patients infectés par le VIH1 ont été évalués. Les caractéristiques sociodémographiques et cliniques des patients sont rapportées dans le Tableau 1. La population était jeune avec un âge médian de 42 ans (IIQ: 36-50), à nette prédominance féminine (79%). Les facteurs de risque cardiovasculaire retrouvés en dehors de l'infection à VIH étaient les dyslipidémies dans 13 cas (10,53%), le tabagisme dans 1 cas (0,8%), l´obésité dans 41 cas (33%), le diabète dans 1 cas (0,8%) et l'hypertension artérielle dans 39 cas (31,7%). La Lipodystrophie était présente dans 8 cas (6,5%). L´infection par le VIH était ancienne comme en témoigne la durée médiane de l´infection à 6,9 années (IIQ: 4,5-9,4). Tous les patients étaient sous thérapie ARV avec une durée médiane d´exposition de 5,3 années (IIQ: 3-7,7) et relativement efficace avec un taux médian de CD4 à 578/mm^3^ (IIQ : 443-758) et une charge virale indétectable chez 82,11% des patients.

**Tableau 1:** caractéristiques sociodémographiques et cliniques des 123 patients infectés par le VIH1 sous traitement antirétroviral suivis à l´hôpital de jour du service des maladies infectieuses du CHUSS de Bobo Dioulasso, novembre 2015

Caractéristiques	N= 123
**Sexe féminin n(%)**	97(78,86)
**Age, médiane (IIQ)**	42ans (36-50)
**Antécédents d´HTA n(%)**	7(5,7)
**Diabète n(%)**	1(0,8)
**Tabac n(%)**	1(0,8)
**Obésité n(%)**	41(33)
**Tour de taille, médiane(IIQ)**	82(75-92)
**Tour de hanche, médiane(IIQ)**	94(88-104)
**Dyslipidémie n(%)**	13(10,57)
**Lipodystrophie n(%)**	8 (6,5)
**HTA n(%)**	39(31,7%)
**Profession femme au foyer n(%)**	46(37,4)
**Taux d´Hémoglobine, médiane (IIQ)**	12,2(10,5-14,7)
**Durée de l´infection VIH, médiane (IIQ)**	6,9(4,5-9,4)
**Temps d´exposition aux ARV, médiane (IIQ)**	5,3(3-7,7)
**Schéma thérapeutique de 1e ligne n(%)**	102(83)
**Taux de CD4 (/mm^3^), (n) médiane (IIQ)**	578(443-758)
**Charge virale indétectable n(%)**	101(82,11)

Les données de l´ECG des 123 patients sont rapportées dans le Tableau 2. Les troubles de la repolarisation étaient les anomalies les plus fréquentes avec 26 cas soit 21,13%. Ils se répartissaient en ischémie sous épicardique dans 20 cas (16,26%), en onde Q de nécrose ancienne dans 4 cas (3,25%) et en lésion sous endocardique dans 2 cas (1,62%). Les autres anomalies observées étaient par ordre de fréquence une onde T inversée isolée en D3 dans 17 cas (13,82%), une onde U dans 15 cas (12,3%), une tachycardie sinusale dans 13 cas (10,57%), une hypertrophie ventriculaire gauche (HVG) dans 12 cas (9,76%) et l´allongement de l´espace QTc dans 7 cas (5,69%). L´HVG était observée chez les patients hypertendus et cinq patients présentant des troubles de la repolarisation étaient hypertendus. L´allongement de l´espace QTc n´était pas statistiquement lié à la prise d´anti protéases (p=1).

**Tableau 2:** données électrocardiographiques des 123 patients infectés par le VIH sous traitement antirétroviral suivis à l´hôpital de jour du service des maladies infectieuses du CHUSS de Bobo Dioulasso, novembre 2015

Données	N=123
**Fréquence cardiaque, médiane (IIQ)**	76(67-88)
**Rythme sinusal n(%)**	123(100)
**Tachycardie sinusale n(%)**	13(10,57)
**Espace PR allongé n(%)**	3(2,4)
**Onde Q de nécrose n(%)**	4(3,25)
**Sous décalage ST n(%)**	2(1,63)
**Ondes T ischémique n(%)**	20(16,26)
**Onde T négative isolée en D3 n(%)**	17(13,82)
**Ondes U n(%)**	15(12,3%)
**HVG n(%)**	12(9,76)
**HVD n(%)**	3(2,44)
**QTc, médiane (IIQ)**	0,38(0,37-0,41)
**QTc allongé n(%)**	7(5,69)

## Discussion

Notre travail qui décrit les anomalies électrocardiographiques chez des patients infectés par le VIH asymptomatique sur le plan cardiovasculaire et suivis en milieu hospitalier montre que ces anomalies sont fréquentes. En témoigne, la prévalence élevée des altérations de la repolarisation avec une prévalence de 21,13% dont 3,25% de nécrose ancienne. Ce constat était déjà rapporté par Sani au Nigéria avec une prévalence de 27% d´altération de la repolarisation [6]. Comparé aux 89% rapportés par Niakara à Ouagadougou [7], notre prévalence semble faible mais cette étude a eu lieu en milieu d´hospitalisation chez des patients infectés par le VIH admis en cardiologie pour des cardiopathies de nature diverse.

L´ischémie myocardique asymptomatique à l´instar d´autres maladies métaboliques comme le diabète de type 2, prémonitoire de la survenue d´évènements cardiovasculaires secondaires est un facteur de mauvais pronostic [8]. De nombreuses études rétrospectives et prospectives ont rapporté un nombre croissant d´infarctus du myocarde chez des patients jeunes (moins de 50 ans) infectés par le VIH et traités par antirétroviraux [4, 9]. Il semble, actuellement, d´après les études les plus récentes [4, 5], que l´incidence de l´infarctus du myocarde dans la population infectée par le VIH soit supérieure à celle de la population générale du même âge, en particulier, chez les patients traités par antirétroviraux d´autant plus que le traitement antirétroviral comporte une antiprotéase. Ce sur risque vasculaire observé chez les patients infectés par le VIH sous ARV s´explique par une accélération de processus d´athérosclérose. Cette athérosclérose accélérée est polyfactorielle associant des facteurs classiques à des facteurs inflammatoires et immunologiques encore mal définis. Plusieurs facteurs classiques peuvent augmenter ce risque: la présence d´une dyslipidémie a été rapportée chez plus de 13% de nos patients infectés par le VIH et traités par antirétroviraux, un diabète (0,8%), le syndrome de lipodystrophie dans 8% des cas. Par contre, le tabagisme retrouvé avec une forte prévalence dans la plupart des études occidentales [1] n´est pas retrouvé dans notre série. Cette différence s´expliquerait d´une part par la forte proportion de femme dans notre série qui culturellement ne fume pas, d´autre part, des sevrages obtenus chez les hommes fumeurs en début de prise en charge de la maladie.

La présentation clinique de la maladie coronaire chez les patients infectés par le VIH semble être similaire à la population générale, allant de l´ischémie myocardique silencieuse à l´infarctus du myocarde avec sus-décalage du segment ST. L´incidence de l´ischémie myocardique silencieuse chez les sujets infectés par le VIH a été évaluée par Duong *et al*. [10] utilisant un électrocardiogramme d´effort simple a montré, parmi 100 sujets infectés, une incidence de 11% de test positif asymptomatique. A la lumière de ses données, et malgré l´absence de tests d´ischémie dans nos conditions de travail, nous pouvons avancer que le risque d´évolution vers la maladie coronaire symptomatique serait une réalité pour nos patients VIH positifs et sous traitement ARV (bien que pour l´instant la majorité soit sous traitement ARV sans anti protéase). La mise en place des tests d´ischémie est ainsi indispensable pour la détection de l´ischémie myocardique silencieuse qui place le patient infecté par le VIH sous ARV dans une logique de prévention secondaire par la mise en œuvre anticipée et renforcée des mesures hygiéno-diététiques et thérapeutiques avec un contrôle strict des facteurs de risque associés.

## Conclusion

Dans cette étude sur les patients infectés par le VIH sous traitement ARV, il ressort que l´ischémie myocardique asymptomatique est fréquente. Il conviendrait de renforcer son dépistage par des tests d´ischémie plus performants pour mieux préciser sa gravité afin d´y apporter une réponse en terme de prévention et de traitement dans cette sous population au risque cardiovasculaire accru.

### Etat des connaissances sur le sujet

- Le traitement antirétroviral est responsable d´une athérosclérose accélérée (maladie coronaire, artériopathie des membres inférieurs, accident vasculaire cérébral);
- Les études épidémiologiques ont montré que l´incidence de l´infarctus du myocarde est plus élevée dans la population VIH que dans la population générale;
- En Afrique la fréquence de la maladie coronaire chez le patient infecté par le VIH est mal connue et quelques rares cas sont rapportés dans des séries hospitalières.

### Contribution de notre étude à la connaissance

- Prévalence de l´ischémie myocardique asymptomatique au Burkina Faso dans la population de patients infectés par le VIH sous traitement antiretroviral;
- Attirer l´attention des médecins prenant en charge les patients VIH sous traitement antiretroviral du risque coronarien dans cette sous population afin de les inciter à rechercher les atteintes ischémiques et placer le patient dans une logique de prévention secondaire;
- Nécessité de mettre en plateau technique adéquat pour la détection de l´ischémie myocardique silencieuse afin de mieux prévenir la maladie coronarienne dans cette sous population.
